# Piperine enhances carbohydrate/fat metabolism in skeletal muscle during acute exercise in mice

**DOI:** 10.1186/s12986-017-0194-2

**Published:** 2017-07-04

**Authors:** Jisu Kim, Kang-Pa Lee, Dae-Won Lee, Kiwon Lim

**Affiliations:** 1 Physical Activity & Performance Institute, Konkuk University, 120 Neungdong-ro, Gwangjin-gu, Seoul, 05029 Republic of Korea; 2 Department of Medical Science, School of Medicine Konkuk University, 120 Neungdong-ro, Gwangjin-gu, Seoul, 05029 Republic of Korea; 3 Department of Bio-Science, College of Natural Science, Dongguk University, Dongdae-ro 123, Gyeongju, Gyeongsangbuk-do 38066 Republic of Korea; 4 Department of Physical Education, Laboratory of Exercise Nutrition, Korea University, 120 Neungdong-ro, Gwangjin-gu, Seoul, 143-701 Republic of Korea

**Keywords:** Acute endurance exercise, Piperine, Carbohydrate metabolism, Fat metabolism, Antioxidant

## Abstract

**Background:**

Exercise promotes energy metabolism (e.g., metabolism of glucose and lipids) in skeletal muscles; however, reactive oxygen species are also generated during exercise. Various spices have been reported to have beneficial effects in sports medicine. Here, we investigated the effects of piperine, an active compound in black pepper, to determine its effects on metabolism during acute endurance exercise.

**Methods:**

ICR mice (*n* = 18) were divided into three groups: nonexercise (CON), exercise (EX), and exercise with piperine (5 mg/kg) treatment (EP). Mice were subjected to enforced exercise on a treadmill at a speed of 22 m/min for 1 h. To evaluate the inflammatory responses following exercise, fluorescence-activated cell sorting analysis was performed to monitor changes in CD4^+^ cells within the peripheral blood mononuclear cells (PBMCs) of mice. The expression levels of metabolic pathway components and redox-related factors were evaluated in the soleus muscle by reverse transcription polymerase chain reaction and western blotting.

**Results:**

There were no changes in the differentiation of immune cells in PBMCs in both the EX and EP groups compared with that in the CON group. Mice in the EX group exhibited a significant increase in the expression of metabolic pathway components and redox signal-related components compared with mice in the CON group. Moreover, mice in the EP group showed greater metabolic (*GLUT4, MCT1, FAT/CD36, CPT1, CS*) changes than mice in the EX group, and changes in the expression of redox signal components were lower in the EP group than those in the EX group.

**Conclusion:**

Our findings demonstrate that piperine promoted beneficial metabolism during exercise by regulating carbohydrate/fat metabolism and redox signals. Therefore, piperine may be a candidate supplement for improvement of exercise ability.

**Electronic supplementary material:**

The online version of this article (doi:10.1186/s12986-017-0194-2) contains supplementary material, which is available to authorized users.

## Background

The western diet generally includes excessive caloric intake. Moreover, many individuals have adopted a sedentary lifestyle, including little or irregular physical activity, leading to increased rates of obesity and mortality [1]. The World Health Organization (WHO) recommends that individuals exercise daily to alleviate obesity and improve health [2]. Therefore, appropriate research is needed to fully elucidate the effects of exercise on health and the appropriate type of exercise that should be adopted to ensure a healthy lifestyle.

Endurance exercise demands consumption of energy through increased metabolism, leading to reduction of body weight [3, 4]. Moreover, endurance exercise involves generation of reactive oxygen species (ROS) during energy synthesis. Excessive ROS generation from high-intensity endurance exercise can lead to muscle rupture and immune system over-reaction [5]. Additionally, continuous exercise increases immune system function by activating immune cells, including T cells, natural killer (NK) cells, and T helper (Th) cells [6, 7]. Despite these findings, additional work is still needed to determine the effects of immune system activation and exercise on the generation of ROS. Low levels of ROS augment metabolism, thereby enhancing cell proliferation and differentiation, whereas the presence of high levels of ROS induces cell death [8, 9]. Enzymes within the redox signaling cascade function to maintain homeostasis despite ROS generation. For example, nicotinamide adenine dinucleotide phosphate (NADPH)-oxidase (NOX), which is encoded by NOX family genes [10], is a key enzyme involved in ROS generation. NOX1 is expressed in a variety of cells and promotes ROS generation [10, 11]. Oxidative stress from the generated ROS is eliminated by antioxidant signaling and enzymes involved in redox homeostasis, such as manganese superoxide dismutase (Mn-SOD), catalase, zinc superoxide dismutase (Zn-SOD), and Ape/Ref-1 [12, 13]. In particular, Ape/Ref-1 is a multifunctional protein with endonuclease, transcription factor, and antioxidant functions. However, the effects of Ape/Ref-1 on the maintenance of redox homeostasis and immune system function in the context of excessive exercise have not yet been elucidated.

Skeletal muscles have important metabolic energy functions based on the generation and utilization of energy sources from the phosphagen system, glycolysis, and the anaerobic system. Carbohydrate and fat metabolism occur in the muscles during exercise [14]. Skeletal muscles generate energy using energy sources source such as glucose, lactate, and fatty acids [4, 15]. These components are transported into muscle cells via specific metabolic transporters, including glucose transporter type 4 (GLUT4), monocarboxylate transporter 1 (MCT1), and FAT/CD36 [16, 17]. The energy required for transport is generated through the tricarboxylic acid (TCA) cycle in the mitochondria [18, 19]. In particular, carnitine palmitoyltransferase 1 (CPT1), which is present in the mitochondrial outer membrane and plays an important role in fat metabolism, transports long-chain acetyl-CoA, which is generated from fatty acids in the inner mitochondrial membrane [20, 21]. In addition, citrate synthase (CS), which converts acetyl-Co-A to citrate before entering the TCA cycle, is also important in the production of energy [22, 23].

In both athletes and individuals who are exercising for the first time, strenuous exercise can cause various types of damage in the body. Therefore, an effective exercise routine for each person is necessary, taking into account nutritional and physiological changes. Accordingly, recent research has focused on the role of health supplements in promoting the beneficial effects of exercise. Piperine, one of the main components of pepper, has been shown to have diverse biological activities, including anti-inflammatory, antioxidant, anti-atherosclerotic anti-obesity effects and blood lipid improvement in a variety of cell types and animal models. However, the effects of piperine on endurance exercise and changes in skeletal muscles have not yet been established.

Accordingly, in this study, we determined the effects of piperine on muscles during endurance exercise in a mouse model.

## Methods

### Animal experiment

Six-week-old male ICR mice (*n* = 18) were purchased from Orient Bio Inc. (Seongnam, Korea). All mice were housed in standard plastic cages under controlled conditions of humidity (50%) and temperature (23 ± 1 °C) with an alternating 12-h light/dark cycle. Mice were acclimated to the laboratory housing conditions for 7 days. Mice were randomized into three groups: nonexercise (CON), exercise (EX), and exercise with piperine treatment (EP). Piperine was dissolved in dimethyl sulfoxide (DMSO; via oral gavage, in a volume of 50 μL) and administered to the EP group at 5 mg/kg; the CON group and EX groups were treated with distilled water via oral administration at 30 min before acute endurance exercise. All experimental procedures were performed at the Animal Experiment Research Center of Konkuk University. This study was conducted in accordance with the ethical guidelines of the Konkuk University Institutional Animal Care and Use Committee. All mice were adapted to treadmill training (Daejong Systems, Korea) at a fixed intensity (10 m/min, 8° slope, 10 min) for 3 days. The following protocols were used: 22 m/min, 8° slope, 60 min (approximately 70% maximal oxygen consumption [VO_2_]) for acute exercise. Blood samples and soleus muscles were collected immediately after exercise.

### Fluorescence-activated cell sorting (FACS) analysis

FACS assays were performed as previously reported [24]. To determine the expression of T-cell-related surface molecules, peripheral blood mononuclear cells (PBMCs) were isolated using a Ficoll-Hypaque gradient (Sigma-Aldrich, UK). For analysis of cytokine production in differentiated Th1, Th2, and Th17 cells, isolated PBMCs were stained with anti-CD3-PerCP, anti-CD4-APC, and/or anti-CD8-APC-Cy7 antibodies (BioLegend, USA). After washing with FACS buffer (0.1% bovine serum albumin [BSA] in phosphate-buffered saline [PBS]), the cells were then stimulated with 50 ng/mL phorbol 12-myristate 13-acetate (PMA; Sigma-Aldrich) and 1 μg/mL ionomycin (Sigma-Aldrich) in the presence of Golgistop (BD Biosciences, USA) for 4 h at 37 °C. The stimulated cells were washed with FACS buffer and fixed for 10 min with 4% paraformaldehyde. After fixation, the cells were permeabilized with FACS Perm 2 according to the manufacturer’s instructions (BD Biosciences, USA) and stained with appropriate fluorochrome-conjugated antibodies, including anti-mouse interferon (IFN)-γ conjugated with fluorescein isothiocyanate (FITC; BD Biosciences, USA) and anti-mouse interleukin (IL)-17A conjugated with phycoerythrin (eBioscience, Germany) antibodies using isotype antibodies as controls. All data were collected on a FACSCalibur flow cytometer (BD Biosciences) and analyzed using FlowJo software (Tree Star Inc., USA).

### Total RNA isolation and reverse transcription (RT)-polymerase chain reaction (PCR)

Total RNA isolation and RT-PCR analyses were performed as previously reported [25]. The soleus muscles of each mouse were harvested, and total RNA was isolated from cells using TRI-reagent (GenDEPOT, Korea). Next, cDNA was synthesized using 1.0 μg of total RNA with oligo dT(18mer). For PCR, 1 μL of cDNA was subjected to predenaturation at 95 °C for 3 min, 35 cycles of denaturation at 94 °C for 1 min, primer annealing at the optimal temperature for 1 min, and extension at 72 °C for 1 min, followed by a final extension at 72 °C for 10 min. The following primers were used for PCR: *GLUT4* (60 °C; sense primer, 5′-AACTTGGCATTGTGGAAGG-3′, and antisense primer, 5′-ACACATTGGGGGTAGGAACA-3′), *MCT1* (60 °C; sense primer, 5′-GCTGGAGGTCCTATCAGCAG-3′, and antisense primer, 5′-AGTTGAAAGCAAGCCCAAGA-3′), *CD36* (60 °C; sense primer, 5′-GGCCAAGCTATTGCGACAT-3′, and antisense primer, 5′-CAGATCCGAACACAGCGTAGA-3′), *CPT1* (60 °C; sense primer, 5′-ATCATGTATCGCCGCAAACT-3′, and antisense primer, 5′-CCATCTGGTAGGAGCACATGG-3′), *CS* (60 °C; sense primer, 5′-CAAGTCATCTACGCCAGGGAC3′, and antisense primer, 5′-CAAAGCGTCTCCAGCTAACCA-3′), and *GAPDH*, sense primer, 5′-GGCATTGCTCCTCAATGACAA-3′, and antisense primer, 5′-TGTGAGGGAGATGCTCAGTG-3′. PCR products were separated by 2.0% agarose gel electrophoresis and visualized by staining with ethidium bromide.

### Tissue processing and immunoblotting assay

Immunoblotting was performed as previously reported [26]. Tissue was homogenized in ice-cold cell lysis buffer (50 mM Tris-HCl, pH 7.4, 150 mM NaCl, 0.25% deoxycholic acid, 1% NP-40, 1 mM ethylenediaminetetraacetic acid [EDTA], and protease inhibitors, including 1 mM phenylmethylsulfonyl fluoride [PMSF], 1 g/mL aprotinin, 1 g/mL leupeptin, 1 mM Na_3_VO_4_, and 1 mM NaF), and homogenates were agitated for 1 h at 4 °C. Homogenates were then centrifuged at 13,000×*g* at 4 °C for 15 min, and the supernatant was collected and stored at 80 °C until further analysis. To analyze protein expression, we performed western blotting using specific antibodies. Briefly, 30 μg of protein from each group was boiled, separated by electrophoresis on 12% acrylamide gels, and then transferred onto polyvinylidene difluoride membranes in transfer buffer at 4 °C for 2 h. The membranes were blocked with 5% BSA in Tris-buffered saline (TBS) at room temperature for 1 h and then washed in TBS with 0.1% Tween 20 (TBS/T). The membranes were incubated overnight at 4 °C with specific antibodies against NOX-1, Ape/Ref-1, Mn-SOD, and β-actin (1:1000 dilution [Santa Cruz, USA]). The membranes were washed with TBS/T, followed by incubation with IgG secondary antibodies conjugated with horseradish peroxidase (1:1000 dilution [Santa Cruz, USA]). The expression levels of the proteins were analyzed using chemiluminescence (ECL Plus Kit; Amersham Pharmacia Biotech). Developed protein bands were visualized and quantified using Image J software (NIH, Bethesda, MD, USA).

### Data analysis

Data were expressed as the mean ± standard error of the means (SEMs). Statistical evaluation of the data was performed using GraphPad Prism, version 5.0 (GraphPad Software, USA). Student’s *t*-tests and one-way analysis of variance (ANOVA) with Tukey’s post-hoc tests were used to compare the data. Differences with *P* < 0.05 were considered statistically significant.

## Results

### Effects of piperine on acute endurance exercise-induced immune responses

**Fig. 1:**
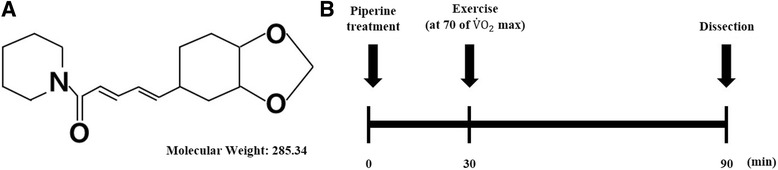
Study design. **a** Structure of piperine (C_17_H_19_NO_3_; molecular weight: 285.34 Da). **b** Study design showing the exercise protocol. Mice were administered 5 mg/kg piperine orally 30 min prior to running on a treadmill for 1 h. After exercise, mice were sacrificed, and tissues were collected

**Fig. 2:**
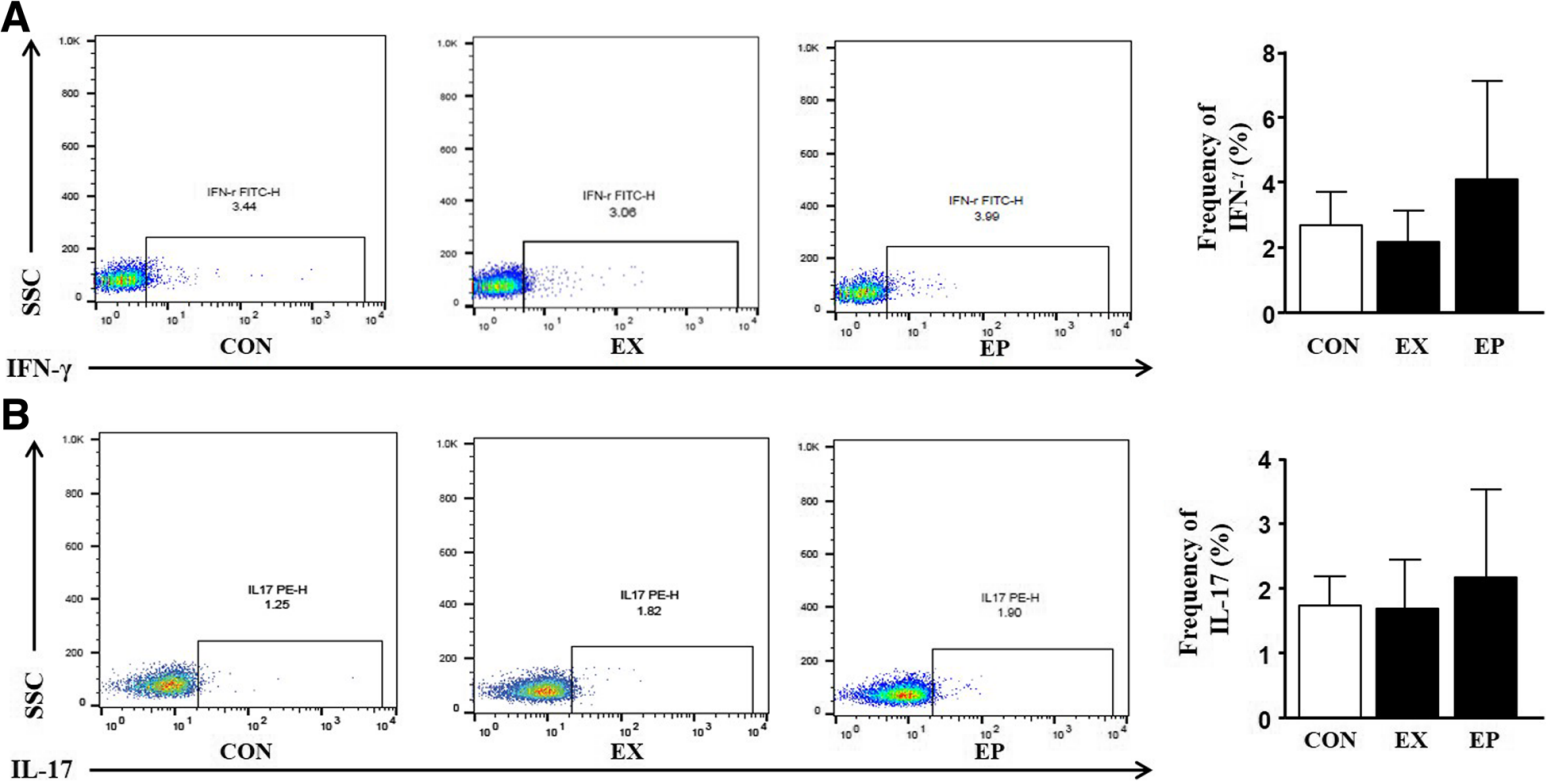
Effects of piperine on the immune response in peripheral blood mononuclear cells (PBMCs) following acute exercise. **a**, **b** Immune cells were prepared from PBMCs. Cells were stimulated with PMA, ionomycin, and golgistop for 4 h and then stained with anti-IFN-γ and anti-IL-17 antibodies for 1 h. Flow cytometry analysis was used to gate IFN-γ^+^ T cells (T helper type 1; Th1) and IL-17^+^ T cells (Th17). The graphs show the frequencies of Th1 and Th17 cells

Physical exercise can enhance the activity of immune responses, including both the innate and adaptive immune responses [27]. Therefore, we investigated the effects of piperine on acute endurance excise by evaluating differentiated Th1, Th2, and Th17 cells in PBMCs using FACS analysis. The one-time acute endurance excise experiment is depicted in Fig. 1b. As shown in Fig. 2, there were no changes in IFN-γ or IL-17 expression in mice in the EX or EP groups compared with those in the control group.

### Effects of piperine on carbohydrate/fat metabolism in soleus muscles from mice after acute endurance exercise

**Fig. 3:**
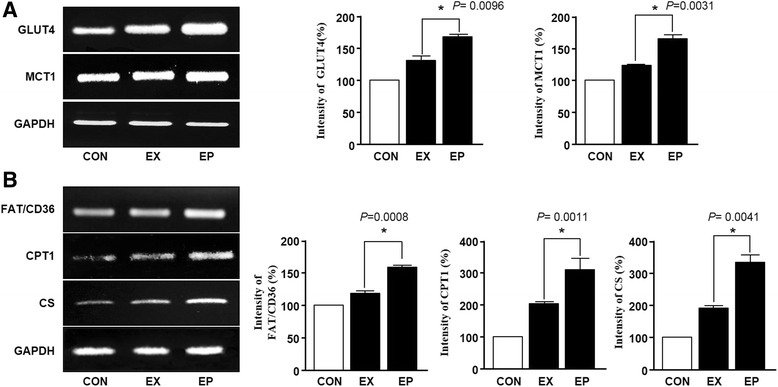
Effects of piperine on the expression of glucose/fat metabolism-associated mRNAs in the soleus muscle after acute exercise. **a** Total RNA from soleus muscles was isolated, and the expression of glucose metabolism-related genes was evaluated using RT-PCR. The graphs were obtained from the left bands and show *GLUT4* and *MCT1* mRNA expression (%). Data are expressed as the mean ± SEM (*n* = 6). mRNA expression in the exercise (EX) group was set at 100%. *P < 0.05 versus the EX group. **b** The expression of fat metabolism-related genes was analyzed by RT-PCR. The graph shows *FAT/CD36*, *CPT1*, and *CS* mRNA expression (%). Data are expressed as the mean ± SEM (*n* = 6). mRNA expression in the exercise (EX) group was set at 100%. *P < 0.05 versus the EX group

The skeletal muscle system represents an accessible metabolic energy source, providing small carbohydrates during exercise [15]. Therefore, to determine the effects of piperine on the energy source used during acute endurance exercise, we evaluated *GLUT4*, *MCT1*, *FAT/CD36*, *CPT1*, and *CS* mRNA expression by RT-PCR. As shown in Fig. 3a, the expression levels of *GLUT4* and *MCT1* mRNAs were higher in the EP group than in the EX and CON groups. Moreover, as shown in Fig. 3b, *FAT/CD36*, *CPT1*, and *CS* mRNAs were also significantly upregulated by exercise and piperine administration. Besides, we performed the following additional experiments. We checked the protein (GLUT4, FAT/36 and CPT1) levels using the western blot assay. As show in Additional file 1: Figure S1, these results showed similar expression of genes and proteins.

### Effects of piperine on antioxidant enzymes in soleus muscles from mice after acute endurance exercise

**Fig. 4:**
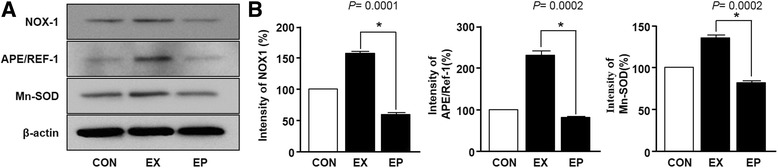
Effects of piperine on the expression of reactive oxygen species (ROS)-regulated mRNA in the soleus muscle after acute exercise. **a** Soleus muscles were collected after exercise, and protein expression was evaluated using immunoblotting. **b** The graphs represent the intensities of NOX1, APE/Ref-1, and Mn-SOD bands (%). Data are expressed as the mean ± SEM (*n* = 6). The band intensity in the exercise (EX) group was set at 100%. *P < 0.05 versus the EX group

High-intensity exercise-induced oxidative stress usually involves elimination mechanisms and homeostasis systems [28]. Therefore, to examine whether piperine inhibited ROS generation, we measured the expression levels of antioxidant enzymes by immunoblotting. As shown in Fig. 4, NOX-1, Ape/Ref-1, and Mn-SOD levels were significantly increased in soleus muscles following exercise as compared with those in the control group. However, piperine administration significantly reduced the expression levels of NOX-1, Ape/Ref-1, and Mn-SOD compared with those in the control group. In addition, we checked the results of L6 cell in vitro as follows. To explore the effect of piperine on H2O2-stimulated L6 cell, we performed the three experiments such as cell observed assay and reverse transcription polymerase chain reaction (RT-PCR) analysis. Cell morphology change was observed by inverted microscope for 1 h, respectively. RT-PCR analysis was used to determine CAT, NOX-1, APE/REF1 and Mn-SOD. As show in Additional file 2: Figure S2A, the cell morphology for 1 h is not altered in all conditions (untreated, 100 μM H2O2, and 100 μM H2O2 with peprine (10 uM). H2O2 significantly increases the expression levels of mRNA CAT, NOX-1, APE/REF1 and Mn-SOD whereas piperine significantly regulated those-signals.

## Discussion

Although piperine has been shown to have anti-inflammatory, anticancer, anti-atherosclerotic, and antioxidant effects and to inhibit lipid synthesis, the effects of piperine on carbohydrate/fat metabolism and skeletal muscle damage during endurance exercise have not been evaluated. Here, we provide evidence of the role of piperine in mediating carbohydrate/fat metabolism in skeletal muscle in acute endurance exercise for the first time. Our findings demonstrate that piperine treatment enhanced fat/carbohydrate metabolism in skeletal muscle and that piperine administration decreased the expression of NOX1 and antioxidant enzymes, such as Mn-SOD and Ape/Ref-1, after acute exercise. Moreover, acute endurance exercise did not alter immune responses, with or without piperine treatment. These results imply that piperine regulated muscle damage and energy metabolism during acute endurance exercise.

Regular exercise can have beneficial effects on health; however, high-intensity physical exercise or excessive exercise can induce muscle degeneration and inflammation [28, 29]. Recently, sports science and nutrition experts have suggested that short-term supplementation with antioxidants may provide beneficial effects on health and reduce damage and inflammation after exercise [30]. Based on our data, there were no changes in immune responses in PBMCs among the three groups (control, exercise, and exercise after piperine administration). These results imply that the performance in the EP group was not related to the innate immune response during acute exercise after short-term administration of piperine. Therefore, we suggest that piperine may be administered safely during exercise.

Exercise is tightly associated with the generation of ROS in the skeletal muscle [30]. During exercise, ROS are produced through energy-generating metabolic processes, which consume high levels of oxygen. We found that NOX1, Mn-SOD, and Ape/ref.-1 expression levels were decreased in mice administered piperine and subjected to acute exercise compared with those in exercised mice without piperine administration. These results imply that piperine provided scavenging activity against superoxide generation, consistent with a previous study [31].

Because of the increased metabolic rate and energy demand associated with exercise, the oxidization of both fat and carbohydrates must be activated simultaneously [4]. Previous studies have reported that FAT/CD36, CPT1, and CS are key components of the molecular machinery required for regulating fat oxidation in skeletal muscle [32, 33]. During exercise, increased glycolysis and subsequent production and accumulation of lactate necessitate increased expression of GLUT4 and MCT1 [16]. Our data also indicate that the expression levels of fat metabolism-related genes, such as *FAT/CD36*, *CPT1*, and *CS*, were significantly increased in the skeletal muscle following exercise with or without short-term piperine administration. A previous study reported that piperine was supplemented in different doses (20, 30 and 40 mg/kg) through administration of a high-fat diet (HFD) for 42 days to experimental rats. Piperine significantly reduced the concentration of plasma and liver lipids in obese rats to near normal levels, and HDL was elevated [34]. Thus, piperine ingestion may influence fat metabolism.

Moreover, our data indicate that the transcription of *GLUT4* and *MCT1* was promoted by acute exercise after short-time administration of piperine. Therefore, we suggest that piperine, an active component of black pepper, may be used as a sports supplement for improving exercise ability and adaption.

However, a limitation of this study is that we did not use the mice only treated with piperine as a control group. This is because we 1) intended to differentiate our study from previous studies and 2) wanted to confirm the effects of supplementation of piperine in acute exercise.

AMP-activated protein kinase (AMPK) is an enzyme that controls key players of metabolic pathways, such as glycolysis and fatty acid oxidation [35, 36]. Although we did not evaluate AMPK signaling in this study, piperine may regulate the AMPK pathway, as shown in previous reports [37] and supported by our current data. Previously, several in vitro and in vivo studies attempted to determine the mechanisms through which piperine affects fat metabolism [38–41]. Overall, our results show that piperine treatment can improve the carbohydrate/fat metabolism in skeletal muscle during acute exercise. Further studies are needed to clarify the long-term effects of piperine on exercise endurance capacity in athletes.

## Conclusion

In conclusion, our findings suggest that piperine improve beneficial energy metabolism during exercise by regulating carbohydrate/fat metabolism without stimulating the innate immune response or superoxide generation. Therefore, piperine may have applications as a nutritional supplement for improvement of exercise ability.

## Additional files

Additional file 1: Figure S1. Effects of piperine on the expression of glucose/fat metabolism-associated protein in the soleus muscle after acute exercise. Total protein from soleus muscles was isolated, and the expression of glucose metabolism-related genes was evaluated using western blot. (JPEG 40 kb)

Additional file 2: Figure S2. Effects of piperine on exogenous hydrogen peroxide (H2O2)-stimulated L6 skeletal muscle cells. L6 cells were treated with piperine (10 μM) and presence or absence of H2O2 (100 μM) for 1 h. These morphological changes were observed by inverted microscope (A). Reverse transcription polymerase chain reaction analysis was used to determine CAT, NOX-1, APE/REF1 and Mn-SOD (B). These graphs are presented as mean ± standard error (*P* < 0.05). (JPEG 230 kb)

## Abbreviations

AMPK: AMP-activated protein kinase
ANOVA: Analysis of variance
CPT1: Carnitine palmitoyltransferase 1
CS: Citrate synthase
FITC: Fluorescein isothiocynanate
GLUT4: Glucose transporter type 4
IFN: Interferon
IL: Interleukin
MCT1: Monocarboxylate transporter 1
Mn-SOD: Manganese superoxide dismutase
NADPH: Nicotinamide adenine dinucleotide phosphate
NK: Natural killer
NOX: Nicotinamide adenine dinucleotide phosphate
PBMC: Peripheral blood mononuclear cell
PCR: Polymerase chain reaction
PMA: Phorbol 12-myristate 13-acetate
ROS: Reactive oxygen species
RT: Reverse transcription
SEM: Standard error of the mean
TBS: Tris-buffered saline
TBS/T: Tween 20
TCA: Tricarboxylic acid
Th: T helper
Zn-SOD: Zinc superoxide dismutase
