# Supplementary figures and images for: Piperine enhances carbohydrate/fat metabolism in skeletal muscle during acute exercise in mice
Source: Nutr Metab (Lond). 2017 Jul 4;14:43. doi: 10.1186/s12986-017-0194-2 (PMC5496355; doi:10.1186/s12986-017-0194-2)

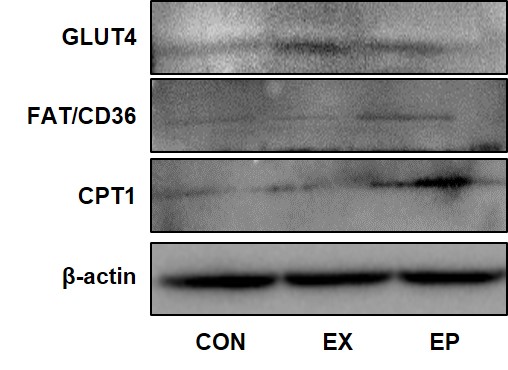

Supplement: Supplementary file 1 — Figure S1. Effects of piperine on the expression of glucose/fat metabolism-associated protein in the soleus muscle after acute exercise. Total protein from soleus muscles was isolated, and the expression of glucose metabolism-related genes was evaluated using western blot. (JPEG 40 kb) [file 12986_2017_194_MOESM1_ESM.jpg]

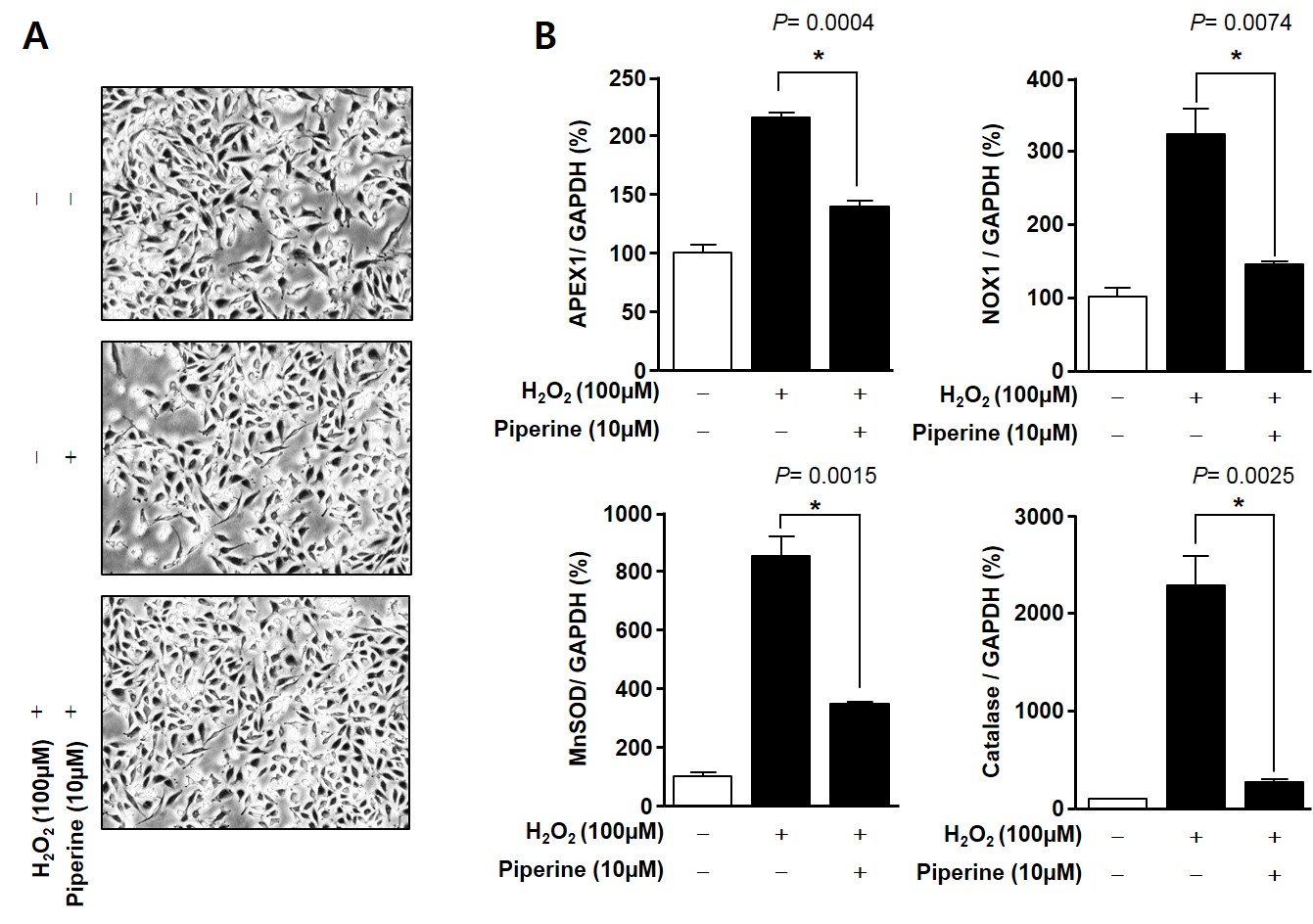

Supplement: Supplementary file 2 — Figure S2. Effects of piperine on exogenous hydrogen peroxide (H2O2)-stimulated L6 skeletal muscle cells. L6 cells were treated with piperine (10 μM) and presence or absence of H2O2 (100 μM) for 1 h. These morphological changes were observed by inverted microscope (A). Reverse transcription polymerase chain reaction analysis was used to determine CAT, NOX-1, APE/REF1 and Mn-SOD (B). These graphs are presented as mean ± standard error (P < 0.05). (JPEG 230 kb) [file 12986_2017_194_MOESM2_ESM.jpg]
